# Biological and physico-chemical properties of new root canal sealers 

**DOI:** 10.4317/jced.54548

**Published:** 2018-02-01

**Authors:** Marco Colombo, Claudio Poggio, Alberto Dagna, Maria-Vittoria Meravini, Paolo Riva, Federico Trovati, Giampiero Pietrocola

**Affiliations:** 1 Department of Clinical-Surgical, Diagnostic and Pediatric Sciences – Section of Dentistry, Policlinico “San Matteo” Piazzale Golgi, Pavia, Italy

## Abstract

**Background:**

The purpose of the present study was to compare the biological and the physico-chemical properties of bioceramic-based root canal sealers, calcium hydroxide-based, MTA-based and epoxy resin-based root canal sealers.

**Material and Methods:**

Two bioceramic-based sealers, one calcium hydroxide-based sealer, one MTA-based sealer and two epoxy resin-based sealers were tested.

**Results:**

EasySeal and MTA Fillapex showed severe citotoxic activity, AH Plus and SealapexTM moderate cytotoxicity, BioRoot™ RCS and TotalFill BC Sealer were both cytocompatible. Except for TotalFill BC Sealer, all root canal sealers caused inhibition zones when tested with E. faecalis. The highest inhibition zone was observed for EasySeal, followed by AH Plus. BioRoot™ RCS, SealapexTM and MTA Fillapex showed the lowest inhibition zone. All the tested materials showed different degree of antibacterial activity by using direct contact test (DCT). The highest values were observed for BioRoot™ RCS, TotalFill BC Sealer and EasySeal, followed by MTA Fillapex and SealapexTM. Except for BioRoot RCS and TotalFill BC Sealer, all the root canal sealers fulfilled the requirements of the ISO 6876 standard, demonstrating a weight loss less than 3%. Bioroot RCS, TotalFill BC Sealer and SealapexTM exhibited high alkaline pH with an increase both for BioRoot™ RCS and TotalFill BC Sealer after 24 hours.

**Conclusions:**

The new bioceramic-based sealers showed acceptable physico-chemical properties, but BioRoot™ RCS and TotalFill BC Sealer seems to be too soluble, not respecting ISO 6876 requirements.

** Key words:**Antibacterial activity, cytoxicity, pH, root canal sealers, solubility.

## Introduction

Root canal sealers are used in endodontics to achieve a stable obturation of the root canal system by creation of a hermetic seal throughout the canal and by the filling of minor incongruities between the dentinal wall and gutta-percha (1) and to entomb bacteria, prevent their ingress from the oral environment and avoid their passage to the periapical tissues (2). An ideal sealer should offer specific properties: tissue tolerance, no shrinkage with setting, slow setting time, adhesiveness, radiopacity, bacteriostatic properties, absence of staining, solubility in solvents, insolubility to oral and tissue fluids (3,4). Insolubility is one of the most desirable physical properties for root canal sealers (5) because it may have a great influence on the success of root canal treatment (2). In fact, the dissolution may cause gaps along the dentin/sealer/gutta-percha interface that might offer a pathway for bacteria and their byproducts into periapical tissues (5,6). Low solubility of a root canal sealer has been introduced in 2000 as a requirement in the ANSI/ADA specification No. 57 (7) and in 2001 as a requirement in the International Standards Organization 6876 standard for root canal sealing materials (8). According to those standards the solubility of a sealer shall not exceed 3% mass fraction after immersion in water for 24 hours (9). In addition, the pH change of sealers may be related with antibacterial outcomes and deposition of mineralized tissue, thus playing a role in the healing process (10-12).

Today different endodontic sealers are available on the market (13). The ZnOE-based sealers have a long history of successful usage, because of their widely demonstrated positive qualities (4). Calcium hydroxide containing sealers supposedly have antimicrobical effects and biologic properties that stimulate a calcific barrier at the apex (4). Amongst resin-based sealers, epoxy-based cements are the primarily ones, with many tested properties like antimicrobial action, adhesion to dentin walls, good seal ability and relative insolubility (4). Because of its favorable biological characteristics, root canal sealers based on mineral trioxide aggregate (MTA) have been introduced (14,15). However, the handling characteristics of MTA preclude the use as a sealer without the addition of chemicals that provide sufficient flow (15). Components such as gels or water-soluble polymers have been added to enhance the cement manipulation (16,17). Various studies reported the biocompatibility of MTA endodontic sealers, which may stimulate mineralization and exhibit bioactivity by stimulating hydroxyapatite nucleation (18). Recently, bioceramic-based sealers containing calcium silicate and/or calcium phosphate have attracted considerable attention because of their physical and biological properties (19,20). They contain calcium phosphate, which improves the setting properties, and offers a chemical composition with crystalline structure similar to tooth and bone apatite materials (21).

The present study studied the biological (cytotoxicity and antibacterial efficacy) and the physico-chemical (solubility and pH) properties of bioceramic-based root canal sealers and compared them to different calcium hydroxide-based, MTA-based and epoxy-resin based root canal sealers.

## Material and Methods

Six different root canal sealers were tested ( Table 1).

**Table 1:**
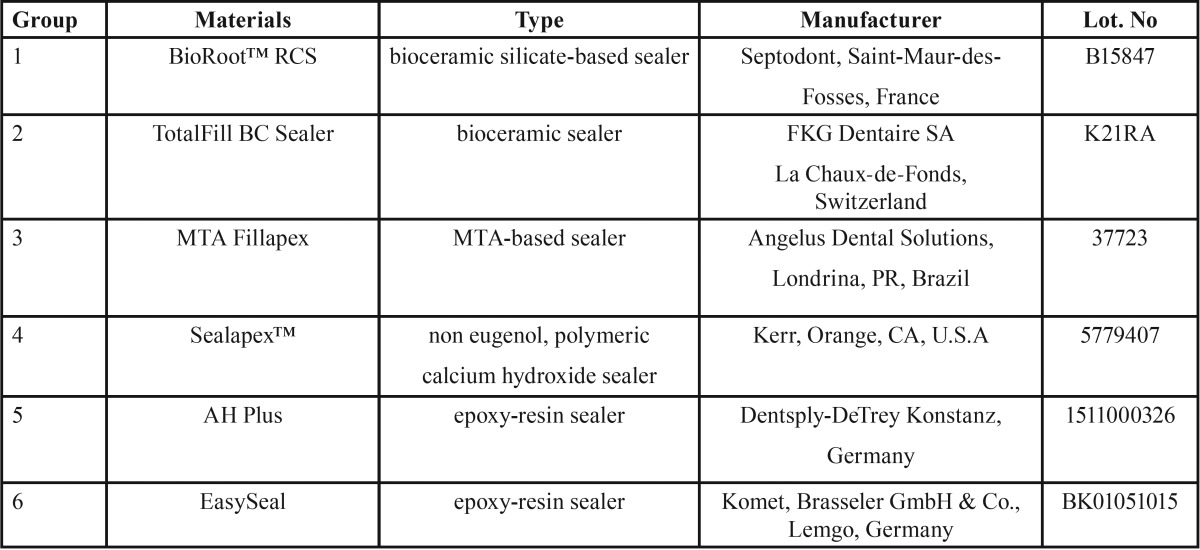
Root canal sealers tested.

-Cytotoxicity assay 

Immortalized human gingival fibroblast-1 HGF-1 (ATCC CRL-2014) were obtained from the American Type Culture Collection. The sealers were placed into sterile, cylindrical Teflon molds and immersed in extraction medium immediately after setting. The extraction was made eluting the sealers in cell culture medium. Cultures were then exposed to 100 μL of the extracts medium. The optical density of formazan dye was read at 545 nm against 620 nm as background by ELISA reader (Bio-Rad, Hercules, California, USA). The percentage of viable cells in each well was calculated relative to control cells set to 100%. Cytotoxicity responses were rated as severe (30%), moderate (30-60%), mild (60- 90%) or noncytotoxic (>90%) (5).

-Antibacterial test 

Agar diffusion test (ADT) was carried out under aseptic conditions in a laminar flow chamber. The antibacterial activity was evaluated using a standard strain of Enterococcus faecalis (ATCC 29212). Four wells for each material were made with a punch by removing the agar at equidistant points and then filled immediately with the materials to be evaluated. The inhibition zones around each one of the wells were then measured in two perpendicular locations with a millimeter ruler with accuracy of 0.5 mm. The size of the inhibition zone was calculated as follows: size of inhibition zone = (diameter of halo – diameter of specimen) x ½ All the assays were conducted in triplicate and the results were recorded in terms of the average diameter of inhibition zone.

Direct contact test (DCT) was used to evaluate the antibacterial properties of the root canal sealers by counting the number of bacterial colonies after plating on agar plates. All sealers were placed in sterile cylinder-shaped plastic blocks and placed in an incubator at 37°C and the humidity of 100% for a period of 7 days. The obtained sealer blocks were grinded and powdered using a ceramic mixer. Equal volumes of bacterial suspension and the sealer suspension (1ml) were mixed. Six, fifteen and sixty minutes after mixing, the suspensions were diluted ten thousand times, and 0.01 ml of the diluted suspension was plated in triplicate on the already-provided BHI agar plates (Difco Lab., Detroit, MI, USA). After incubation at 37°C for 24 h, the colonies formed on the agar plated were counted. Then, the number of colony-forming unit (CFU) was calculated for the different times of the experiment.

-Solubility evaluations 

The solubility was determined respecting the International Standards Organization (ISO) 6876 (7) method and the American Dental Association (ADA) specification No. 57 (8). Stainless steel ring molds with an internal diameter of 20 ± 0,1 mm and a height of 1,5 ± 0,1 mm were used for sample preparation. All molds were weighted 3 times before use (accuracy ± 0,0001 g) using a precision balance (Mettler-Toledo, model AE1633, Novate Milanese, Italy). The molds were placed on a glass plate and filled to slight excess with the mixed materials. The difference between the final mass and the initial mass divided by the initial dry weight of the sample x 100, correspond to the loss of mass of each specimen express as percentage of solubility (9,22). The solubility test was repeated 2 months after by using the same method (9). The solubility of the root canal sealers should not exceed 3 % mass fraction (ISO 6876 clause 4.3.6).

-pH measurements 

Each root canal sealer was placed onto cylindrical Teflon molds. The samples were allowed to set in a cabinet (37˚C, > 95% relative humidity). Each sample was placed into a separate vial, containing 10 mL distilled water. The samples were stored at 37°C, and pH measurement was performed 3 and 24 hours after incubation. The pH value was measured by a digital pH meter. Six samples were prepared for each group and Tukey’s test was applied to determine whether significant differences existed in pH values after 3 hours of incubation. To determine whether time influenced the pH values of the pulp capping materials, an analysis of longitudinal data was performed using t-test for paired data (*P*<0.05) between times of incubation (3 and 24 hours). Statistical analysis Data collected were analyzed with Prism 4.0 (GraphPad). A significant difference between the various groups in the ADT was determined with the Mann-Whitney U-test. Significant differences between the groups in the DCT were identified by multiple comparisons according to Bonferroni two-tailed t-test for independent random samples. Data obtained from solubility test were analyzed with Mann-Whitney U-test. To determine whether time influenced the solubility of the pulp capping materials, an analysis of longitudinal data was performed using t-test for paired data (*p*<0.05). Tukey’s test was applied to determine whether significant differences existed in pH values after 3 and 24 hours of incubation. Significance for all statistical tests was predetermined at *p*<0.05.

## Results

-Biocompatibility 

**Figure 1:**
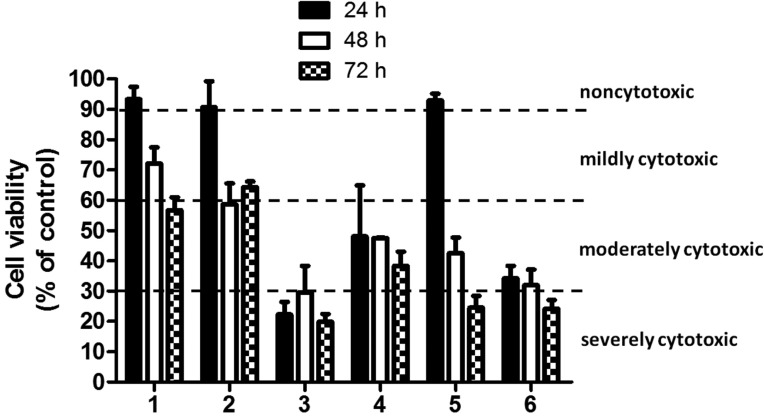
Cell viability in the presence of the elute extracts from eight root canal sealers. Confluent human gingival fibroblasts were treated for 24 hours with extracted medium made eluting the sealers for 24 hours, 48 hours or 72 hours. The cell viability was measured by the MTT assay. Values are expressed as percentages relative to the control group and classified as severe (<30%), moderate (<60%), mild (60-90%) or non-cytotoxic (>90%). Bars and error bars represent the means and ± SD from three independent determinations performed in triplicate.

The results are shown in  Table 2 and represented in Fig. 1. BioRoot™ RCS and TotalFill BC Sealer extracted for 24h showed no cytotoxic effect, while it was mild by using 48 and 72h extracts. Differences in cytotoxicity for all the times were not statistically significant (*p*>0.05). No cytotoxic effect was measured by using AH Plus medium eluted for 24 h, while it was moderate after 48 h and severe after 72 h. Sealapex™ showed moderately cytotoxic activity for all the extraction times. EasySeal and MTA Fillapex remained severely or borderline mildly cytotoxic for all the extraction times. After 72h of elution, both sealers exhibited a toxicity level that was significantly more severe (*p*<0.05) than the other tested sealers.

**Table 2:**
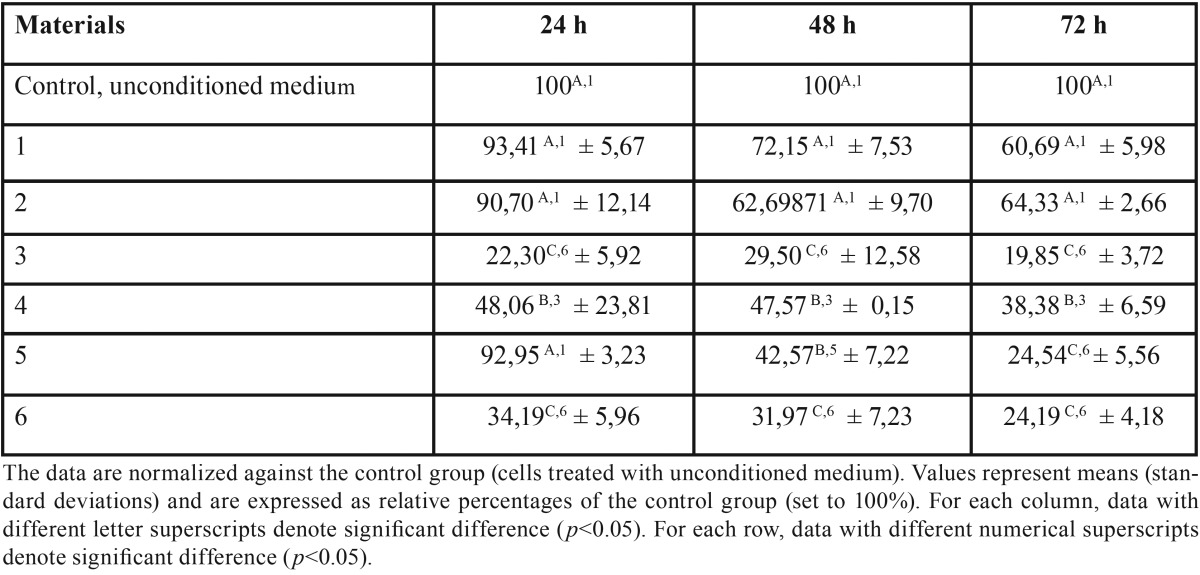
Cell viability in the presence of the eluate extracts from six root canal sealers.

-Antibacterial activity 

**Figure 2:**
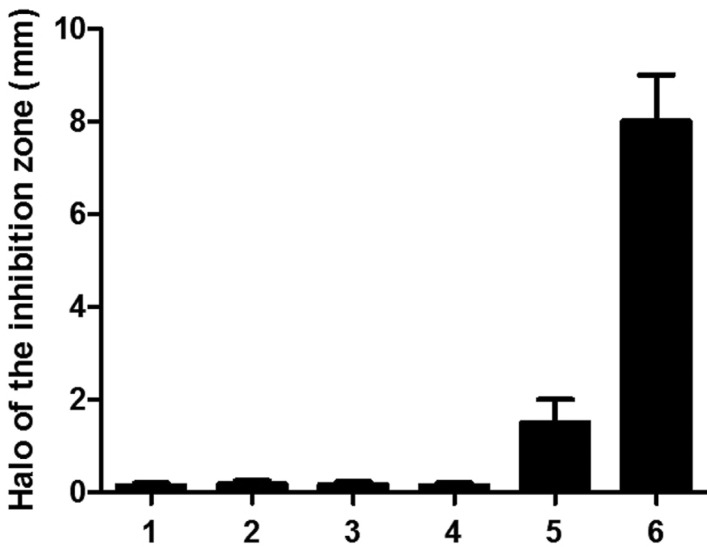
Antibacterial activity of the different pulp canal sealers evaluated by agar diffusion test. 5 mm in diameter and 2 mm deep disks composed of each pulp canal sealers were placed on agar plates previously incubated with incubated with Enterococcus faecalis and incubate at 37°C for 24h. All the assays were conducted in triplicate and the results were recorded in terms of the average diameter of inhibition zone (mm). Error bars indicate standard errors of the means. Statistically significant differences are indicated (Student’s t test; * *P*<0.05; **P<0.01).

The results of the Agar diffusion test (ADT) are shown in  Table 3 and represented in Fig. 2. The mean diameter of the bacterial inhibition zone by freshly mixed EasySeal sealer (8.10 ± 0.2) was statistically different compare to the others (*P*<0.01). AH plus sealer showed lower antibacterial activity as reflected by measuring the inhibition zone (1.2 ± 0.2). However, significant differences between the inhibition zone by EasySeal and AH Plus sealers were shown (*P*<0.05). BioRoot™ RCS, MTA Fillapex and Sealapex™ showed the lowest antibacterial activity compared to the others (bacterial inhibition zone; 0.2 ± 0.05, 0.3 ± 0.02 mm or 0.2 ± 0.04 respectively).

**Table 3:**
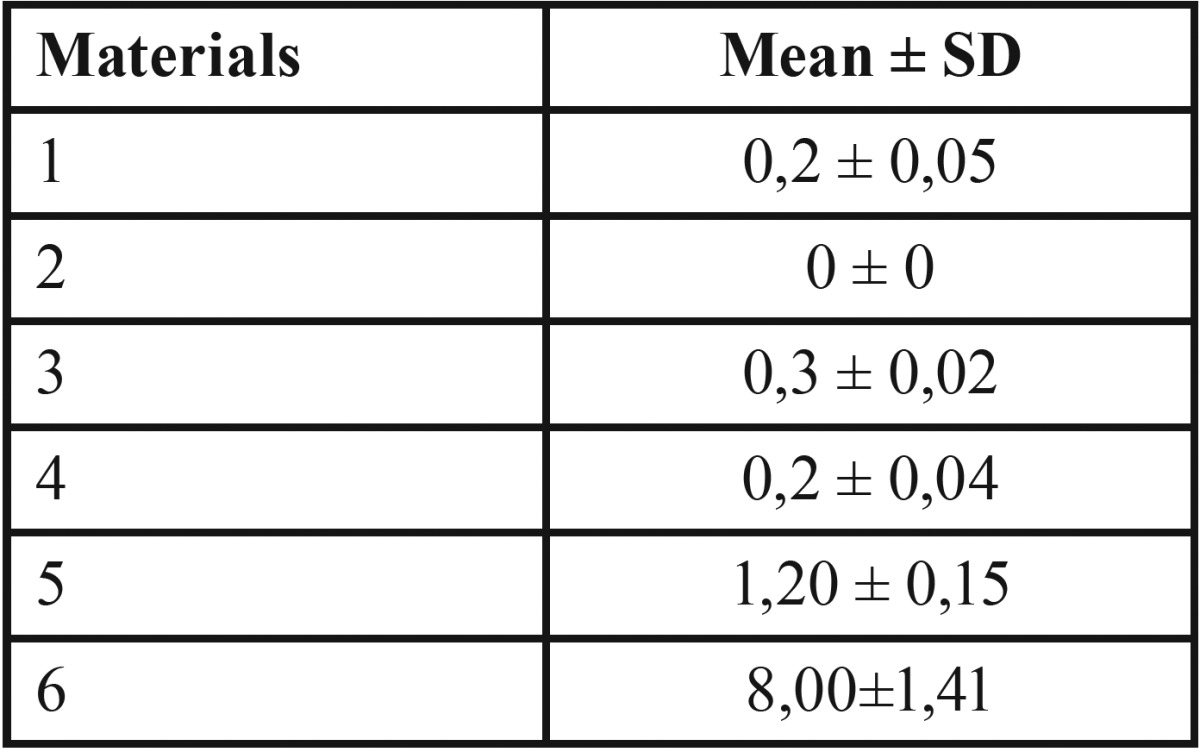
Mean diameter ± standard deviation (mm) of the bacterial inhibition zone by pulp canal sealers evaluated after 48h by ADT. 5 mm in diameter and 2 mm deep disks composed of each pulp canal sealers were placed on agar plates previously incubated with Enterococcus faecalis at 37°C for 24h. All the assays were conducted in triplicate and the results were recorded in terms of the average diameter of inhibition zone (mm).

**Figure 3:**
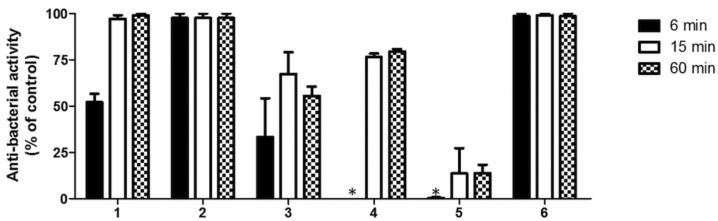
Antibacterial activity of the endotontic sealers at different experimental times on *Enterococcus faecalis* by direct contact test. Antibacterial activity is expressed as percentage of that observed in the absence of the sealer (0%). The data points are the means +/- SD of three independent experiments each performed in triplicate. Asterisk (*) indicates no statistically significant differences between the bacterial cells treated with sealer saline suspension or the sealer-free saline suspension (control).

The results of the Direct contact test (DCT) are shown in Fig. 3 and express as percentage of antibacterial activity compare to the negative control. All sealers were distinctly different from each other in their antimicrobial activity. Sealapex™ and AH Plus doesn’t show any bactericidal effect after 6 min of contact. After 15 and 60 min of contact a significant increment (*P*<0. 01 for Sealapex™ and *P*<0. 05 for AH Plus) of the bactericidal effect was found (Fig. 3). Significantly higher was the antibacterial effect of Sealapex Root Canal Sealer compare to that observed for AH Plus (*P*<0.01). BioRoot™ RCS and MTA Fillapex showed at least means (4 ± 2 x 107/ml) of the number of colonies formed in milliliter after 6 min of contact. A significant increase in bactericidal effect (*P*<0.05) after 15 and 60 min for BioRoot™ RCS and MTA Fillapex was found. For every contact times considered, both TotalFill BC Sealer and EasySeal were bactericidal against *E. faecalis* and killed all bacteria.

-Solubility evaluation 

The results are listed in  Table 4. Both BioRoot™ RCS and TotalFill BC Sealer showed significantly higher (*P* < 0.05) solubility among the tested materials; although the highest solubility percentage was recorded for TotalFill BC Sealer. For remnant materials analized (MTA Fillapex; Sealapex™; AH Plus and EasySeal) fulfilled the requirements of the International Standard Organization 6876 (7) and ANSI/ADA specification No. 57 (8), demonstrating a weight loss of less than 3%.

**Table 4:**
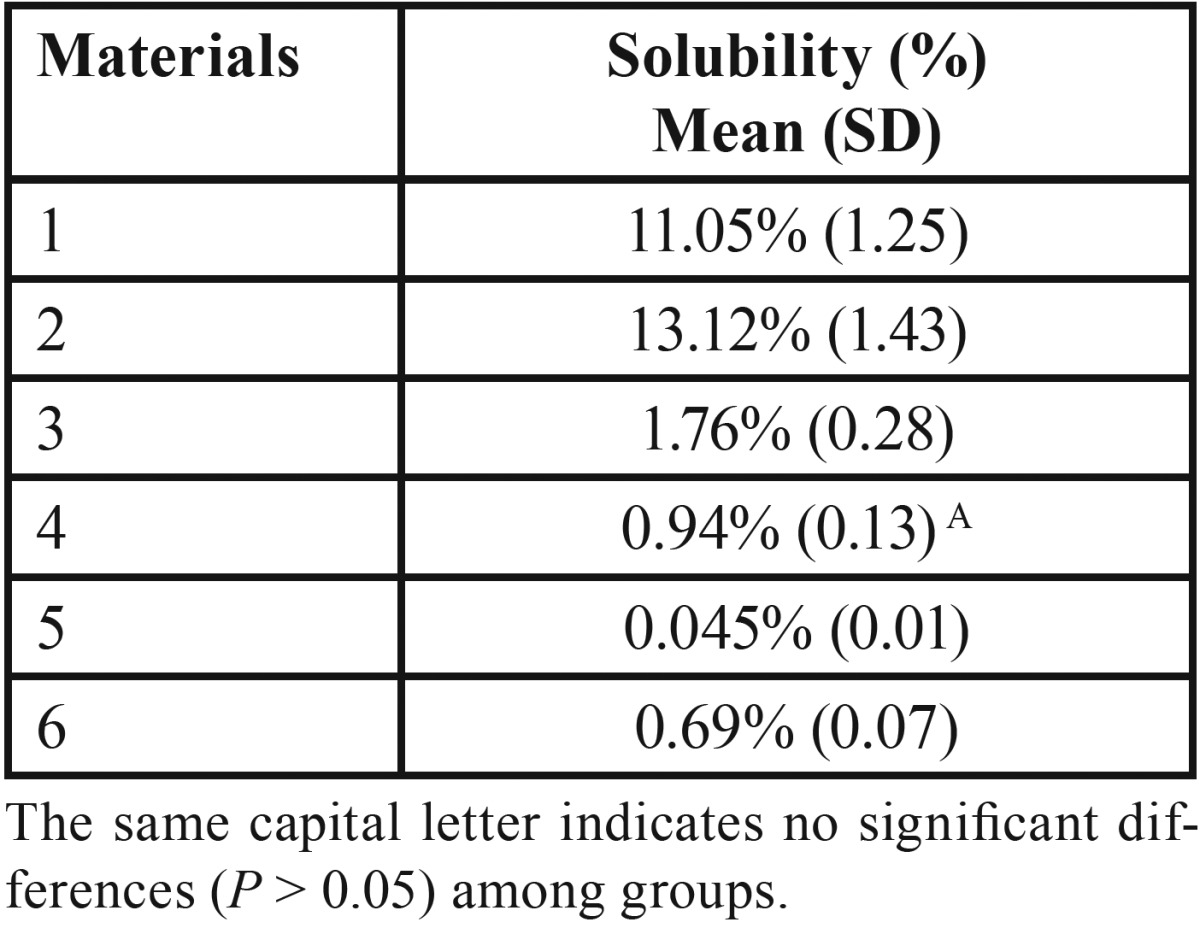
Mean percentage values of solubility and standard deviation (SD) for each material.

-pH measurement 

The pH mean values of all tested materials at different immersion times (3 and 24 h) are described in  Table 5. Bioroot™ RCS, TotalFill BC Sealer and Sealapex™, exhibited high alkaline pH over time; although the significantly highest alkaline pH was recorded for TotalFill BC Sealer (*P* < 0.05). No significant variation in pH was observed for Sealapex™ over time, whereas it was significant for both BioRoot™ RCS and TotalFill BC Sealer (*P* < 0.05). Significantly lower (*P* < 0.05) was the alkalinity of EasySeal, MTA Fillapex and AH Plus than that observed for BioRoot™ RCS, TotalFill BC Sealer and Sealapex™. MTA Fillapex exhibited an initial neutral pH (7.68) that was followed by a weak alkaline pH (8.02). Whereas, AH Plus had an initial weak alkaline pH (8.0) followed by a neutral pH (~7.6).

**Table 5:**
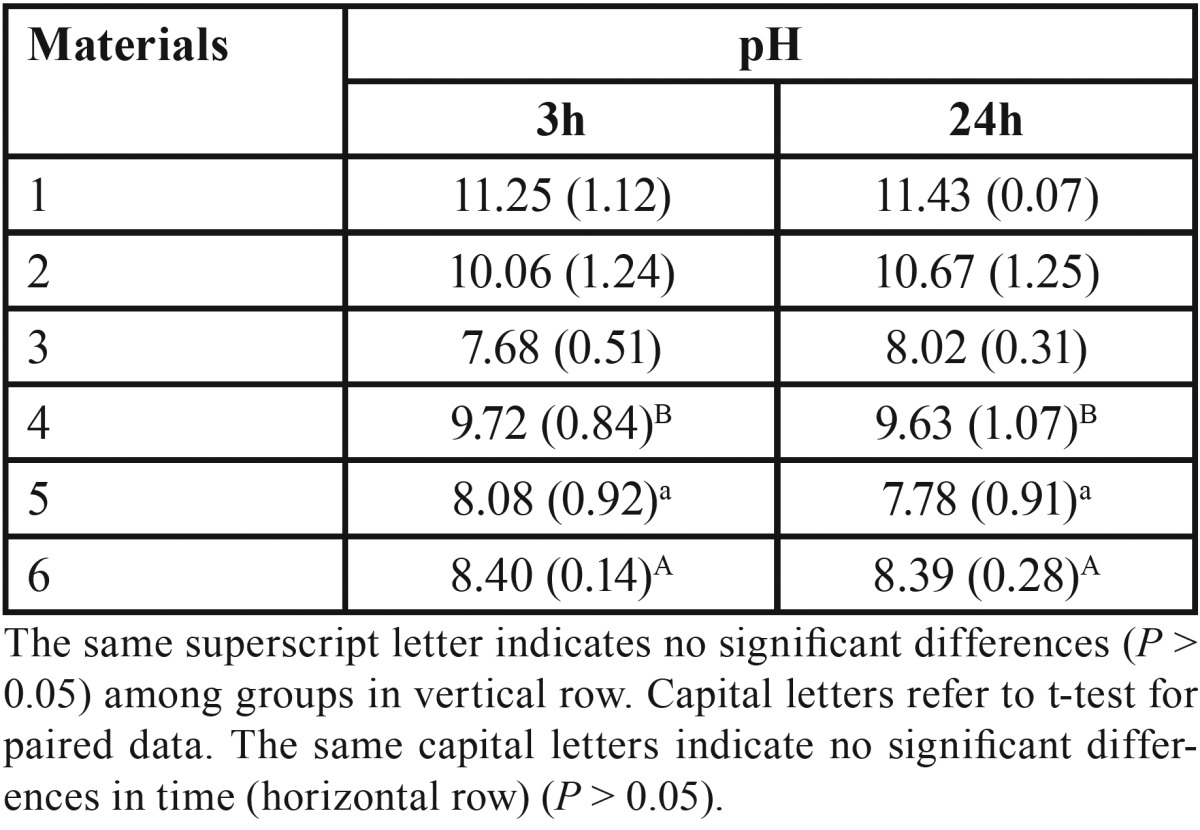
Mean pH values and standard deviation (SD) for each the tested materials at 3 and 24 after incubation.

## Discussion

The first results of this study revealed that EasySeal and MTA Fillapex have a greater cytotoxic effects while AH Plus and SealapexTM moderate cytotoxicity. Cytotoxic effects of MTA Fillapex have been previously documented in Literature (25): some components, like salicylate resin, diluting resin, and silica, may explain these results. Moreover, MTA Fillapex probably has an unbalanced ratio among resin and MTA, with higher values for salicylate resin.

BioRoot™ RCS and TotalFill BC Sealer showed no cytotoxic effects under the present experimental conditions and discrete antibacterial activity, both for Agar diffusion test (ADT) and direct contact test (DCT) against E. faecalis Osteogenic potential, biocompatibility, and antibacterial ability are related to alkaline pH, which can neutralize the lactic acid from osteoclasts and prevent dissolution of mineralized components of teeth (23). Therefore, root canal sealers can contribute to hard tissue formation by activating alkaline phosphatase (23). In this study the alkalinity of EasySeal, MTA Fillapex and AH Plus was significantly lower (*P* < 0.05) than that observed for BioRoot™ RCS, TotalFill BC Sealer and Sealapex™. The pH value of bioceramic-based root canal sealers remained higher than that of epoxy resin-based sealers.

For the pH measurements, all cements were immersed in water immediately after manipulation and they showed a tendency to a reduction in their ability to raise the pH of most materials between 3 and 24 hours. BioRoot™ RCS, TotalFill BC Sealer and Sealapex™ exhibited high alkaline pH over time. Setting and solubility are also important to provide adequate working time and proper consistency enough to seal the root canal system completely (23).

Solubility is the mass loss of a material during a period of immersion in water (24). According to ANSI/ADA Specification 57, the solubility of a root canal sealer should not exceed 3% by mass. A highly soluble root canal sealer would invariably permit the formation of gaps within and between the material and the root dentin, thereby providing avenues for leakage from the oral cavity and periapical tissues.

In the present study BioRoot™ RCS and TotalFill BC Sealer showed significantly higher (*P* < 0.05) solubility among the tested materials; although the highest solubility percentage was recorded for TotalFill BC Sealer. For remnant materials analized (MTA Fillapex, Sealapex™, AH Plus and EasySeal) fulfilled the requirements of the International Standard Organization 6876 (7) and ANSI/ADA specification No. 57 (8), demonstrating a weight loss of less than 3%.

The Literature contains conflicting accounts, with Viapiana *et al.* (1) finding root canal sealers like MTA Fillapex to be highly soluble and Vitti *et al.* (26) reporting the solubility of MTA Fillapex to be <3%, consistent with ISO 6876/2001. This discrepancy between the findings of these studies might be attributed to variations in the methods used to dry the samples after having subjected them to solubility testing.

## Conclusions

Based on the present results, within the limitations of this study, the new bioceramic-based sealers showed acceptable physicochemical properties and promising results as root canal sealers but BioRoot™ RCS and TotalFill BC Sealer seems to be too soluble, not respecting ISO 6876 requirements. For this reason they do not fulfill all of the requirements demanded of the ideal root sealer. Further studies are required to clarify the clinical outcomes associated with the use of these sealers.
